# How did the first COVID-19 lockdown affect persons with concurrent mental health and substance use disorders in Norway? A qualitative study

**DOI:** 10.1186/s12888-022-03812-7

**Published:** 2022-03-14

**Authors:** Marja Leonhardt, Morten Brodahl, Nicola Cogan, Lars Lien

**Affiliations:** 1 Norwegian National Advisory Unit on Concurrent Substance Abuse and Mental Health Disorders, Innlandet Hospital Trust, Post Box 104, 2381 Brumunddal, Norway; 2 Faculty of Health Studies, VID Specialized University, Oslo, Norway; 3 School of Psychological Sciences & Health, University of Strathclyde, 40 George Street, G1 1QE Glasgow, UK; 4 Department of Health and Social Science, Inland Norway University of Applied Science, Elverum, Norway

**Keywords:** COVID-19, Lockdown, Substance use disorder, Mental health disorder, Thematic analysis, Qualitative study

## Abstract

**Background:**

The outbreak of COVID-19 with its severe social restrictions touched the daily life of most people. While everyday social life becomes difficult for citizens with economic and cultural capital, it becomes even worse for vulnerable groups such as persons with mental health and substance use disorders, who are particularly vulnerable to social exclusion. The aim of this study is to investigate how the first COVID-19 lockdown affected the everyday life and health of persons with co-occurring mental health and substance use disorders.

**Methods:**

This qualitative study reports data from 17 individual interviews and one focus group of five participants, all with a self-reported mental health and substance use disorder. Interviews were conducted based on a semi-structured interview guide in September and October 2020 in a medium-sized local authority in Norway. Data were analysed using thematic analysis. A reference group of people with varied knowledge and experiences of the phenomenon were involved in study design, recruitment, data generation and analysis.

**Results:**

The analysis identified four interrelated main themes, describing how the first lockdown affected the everyday life and health of persons suffering from a mental health and substance use disorder: (1) The COVID-19 outbreak as a perceived challenge, (2) A decline in mental health and well-being, (3) Increased substance use challenges, and (4) Diverse experiences with health and social services. The results show that people with a co-occurring disorder have challenges with digital tools and/or do not have the appropriate equipment. Further, participants were not concerned about becoming infected themselves, but infecting others.

**Conclusions:**

Persons with a mental health and substance use disorder face major challenges during the COVID-19 pandemic. There is a need to maintain continuous low-threshold services especially directed towards persons with co-occurring disorders during the pandemic. Furthermore, it is important to improve the digital skills of every service user or offer alternatives to digital consultations and meetings.

## Introduction

There is increasingly strong evidence that public and individual mental health have been affected by the COVID-19 pandemic [1]. The uncertainty of the pandemic, with its lockdowns, physical distancing and related containment strategies, and the resulting impact on the economy and health care, have reportedly increased the risk of mental health problems in the general population and exacerbated health inequalities [2, 3]. Existing disparities in service access are thought to have been widened by the pandemic. Persons with concurrent mental health disorders (MHD) and substance use disorders (SUD), who are often subjected to social exclusion [4], are a particularly vulnerable group during pandemics and lockdowns. Barriers to accessing health care during pandemic outbreaks places persons with MHD/SUD at greater risk, as health care services are pushed to their capacity. People with MHD/SUD, already stigmatised and underserved by the health care system [5], have experienced even greater barriers to treatment for COVID-19 and other diseases during the lockdown [6]. These barriers also apply to testing and imply a greater risk of spreading the virus among people with MHD/SUD [7, 8]. Further, lockdowns and other public health measures also disrupt access to syringe services, medications, and treatment and support services such as opioid substitution treatment (OST). This is likely to have a major impact on persons with MHD/SUD, as they are dependent on continuity of care and support from people they trust [9, 10]. The Norwegian government announced a national lockdown on 12th of March 2020 and the stepwise lifting of restrictions from the 7th of May 2020. The national regulations under the first lockdown included restrictions on domestic and international travelling, the closure of schools and academic institutions and gathering of more than five persons was prohibited. Further, cultural and sports events, gyms and on-to-on-services like hairdressing, skincare, massage, body care and tattooing were banned. Swimming pools and other recreation facilities were closed and restaurants, coffee shops and pubs, if social distancing between customers was not maintained [11]. Violations were punishable by fines or imprisonment for up to six months. As another consequence of infection control measures implemented at population level, low-threshold facilities for persons with MHD/SUD were closed during the first lockdown and many MHD/SUD patients who were admitted to long-term inpatient care were discharged. Non-COVID-19 hospital treatment was deprioritized, and public health nurses and social workers were moved out of protective services and into infection tracing. Persons in institutions like nursing homes, supported housing facilities, psychiatric institutions or prisons have also been deprived visitation rights [11]. In the two largest cities in Norway, isolation units for COVID-19-positive persons with a SUD were established [12] and some NGOs made an effort to distribute injection equipment and food in the streets, but this was not implemented on national level. According to the Church City Mission and several other NGOs in the field of mental health and substance abuse, these measures might have had major consequences for the relapse rate and further reduce the quality of life of persons with MHD/SUD [13]. As pre-existing MHD and SUD are likely to worsen in the face of fear and distress as in a pandemic outbreak, lockdowns imply an even greater burden for persons with MHD/SUD [14]. They are more likely to experience homelessness or incarceration [15] and they face novel challenges, as many of the low-threshold public meeting places are closed and the possibility to access other forms of services is limited. There were no indications during lockdown of any shortage of illegal drugs.

Statistics from the Norwegian “Service User Plan 2019” have identified approximately 26 000 persons in Norway with addiction and mental health problems who have low levels of functioning in important areas of life, such as housing, finances, social life and integration in their communities [16]. Their living standard index has shown gradual deterioration over a period of four years. About 12% have little or no contact with social networks of any kind, are isolated or have only marginal social relationships, 60% have little or no meaningful activity and 17% are homeless. They are often referred to as the “clients in greatest need” [17]. Individuals with MHD and SUD are particularly affected by social exclusion and often exposed to stigma, which may lead to limited utilisation of addiction and mental health services and other health services [18, 19], which are of great importance in a pandemic outbreak such as that of COVID-19. Yet we still know little about how people with MHD/SUD are affected by the COVID-19 pandemic. There is limited research on persons with concurrent MHD and SUD in general, and current research mostly focuses on persons who develop MHD/SUD as a consequence of a pandemic [20, 21]. Although the effects of pandemics such as COVID-19 on persons with MHD/SUD have not been systematically studied, we anticipate that the pandemic will have major consequences for these persons, especially in view of current public reactions. Thus, this study aims to explore how the first COVID-19 lockdown affected the everyday life and health of persons with concurrent MHD and SUD. The study focuses on the social dimensions of the pandemic, such as investigating how adults with MHD/SUD perceived the COVID-19 pandemic in its early stages. The present analysis is the qualitative part of the project “Impact of the COVID-19 outbreak on persons with concurrent mental health and substance use disorders” which also includes a register study. In the qualitative study we are generating data to inform measures to enhance social integration for and resilience in persons with MHD/SUD affected by the COVID-19 lockdown.

## Method

### Design

This qualitative study used a descriptive and exploratory design, with an inductive approach [22]. We have chosen a design with individual interviews and focus groups to get a rich material and to give our target group the possibility to choose their preferred setting as the group is heterogeneous in terms of the societal and health-related challenges. Further, Guest et al. [23] found that sensitive and personal disclosures were more likely to arise in a focus group setting, and that some sensitive themes only occurred in the focus group context. On the other hand, individual interviews allow for deeper reflections and are suitable for people who feel uncomfortable talking in a group [24].

Based on a study on collaborative research by Moltu et al. [25], we established a reference group that supported the authors throughout the research process. Besides the authors, the reference group consisted of representatives of Non-governmental user organisations in the field of MDH/SUD who have lived experience of co-occurring disorders and health care workers. It has been shown that involving people with first-hand experience of the condition in an investigation can increase the quality, relevance and utility of the study findings [26]. These people participated in developing the interview guide, defining the inclusion criteria and the recruitment strategy, and analysing the data. One peer support worker helped to conduct the interviews and was a co-author.

### Recruitment

We used a criterion-based sampling procedure in the recruitment of a heterogeneous sample of 25 persons of both sexes with MHD/SUD with substantial impairment in the last two years, receiving local services in a medium-sized local authority in Eastern Norway. Inclusion criteria were: (a) age 18 years and above, (b) capacity to understand study information and informed consent, (c) being in contact with primary health and social services (e.g., receiving social welfare, registered in an opioid substitution program and/or accommodated in social housing), and (d) having a mental health and substance use problem that seriously affected everyday life, based on self-report. Inclusion did not depend on a clinical diagnosis, as. we did not want to exclude those individuals who may not have formally sought help through mental health and/or addiction services (e.g. due to attitudinal or structural barriers in accessing services and stigma in help seeking) as they represent some of the most vulnerable and ‘easy to ignore’ populations within society. Participants were recruited in cooperation with peer support workers, primary health and social services, a non-governmental organisation (NGO) which provides a low-threshold service for persons in addiction, and FACT (Flexible Assertive Community Treatment) teams in two medium-sized local authorities in eastern Norway. The staff at the NGO, the health and social services, and the peer support workers invited eligible service users to participate in the study. Every person who was interested in participating was then called by the first author and assessed in relation to the inclusion criteria. Finally, an appointment for an interview was agreed where the written informed consent was obtained.

### Participants

The study population comprised 15 men and 7 women of Norwegian origin who reported coexisting mental health and substance use problems (*N* = 22). As mentioned, we aimed, to interview around 25 persons to reach saturation, i.e. a point at which no new information emerged and the content became repetitive, yet with enough depth for the thematic analysis [27]. After conducting 17 individual interviews and one focus group, we reached saturation of general information when no new obvious facts and experiences regarding the first period of the COVID-19 pandemic emerged. Participants were between 24 and 65 years old and used mostly amphetamines, heroin and alcohol, a few smoked cannabis, while six people were receiving OST and three persons indicated that they had been abstinent for several months from heroin. The most reported mental health problem was depression, followed by attention deficit/hyperactivity disorder (ADHD) and anxiety and as well as post-traumatic stress disorder (PTSD). One person was living with parents while one participant reported that the only choice was to live homelessly. About half of the informants lived in supported housing, which means that approximately five persons share a house where everyone has their own room with kitchen and bathroom facilities, supported by inhouse health and social workers. The other informants were living in council accommodations, which is partly or fully financed by the municipality. All participants were either in contact with the local council, mental health and/or addiction services, or receiving services from the FACT team. Most interviewees also had self-reported physical disorders such as chronic obstructive pulmonary disorder (COPD), diabetes or musculoskeletal disorders. None of the participants had tested positive for the coronavirus SARS-CoV-2. The characteristics of the participants are presented in Table 1.

**Table 1:** Characteristics of the study population

Gender	Age	Mental health problem	Substances used	Physical disorders	Treatment
Thale (f)	60	Depression	Alcohol, OST	Diabetes	Local MH/ addiction services
Bjørn (m)	41	Anxiety	Amphetamine, Heroin		Local MH/ addiction services
Vegard (m)	38	ADHD	Amphetamine, Heroin		Local MH/ addiction services
Hans (m)	39	PTSD	Alcohol OST		Local MH/ addiction services
Espen (m)	31	Anxiety	Alcohol Amphetamine Heroin		Local MH/ addiction services
Marius (m)	39	Depression	Amphetamine, Heroin		Local MH/ addiction services
Kari (f)	56	Depression	Abstinent		Local MH/ addiction services
Mari (f)	47	Anxiety	OST		Local MH/ addiction services
Jens (m)	57	Depression	Cannabis	COPD	Local MH/ addiction services
Hilde (f)	60	Depression	Amphetamine	COPD	Local MH/ addiction services
Kevin (m)	47	Depression	Alcohol, Cannabis	COPD	Local MH/ addiction services
Harald (m)	43	Anxiety	Alcohol	Musculoskeletal disorders	Local MH/ addiction services
Elisabeth (f)	34	ADHD	Amphetamine Heroin		Local MH/ addiction services
Nina (f)	23	PTSD, Depression	Amphetamine Cannabis OST		FACT
Ragnhild (f)	54	ADHD	OST	COPD, Diabetes	Local MH/ addiction services
Tuva (f)	46	ADHD	OST		FACT
Steffen (m)	45	ADHD	Alcohol, Cannabis Heroin	Musculoskeletal disorders	Local MH/ addiction services
Peter (m)	50	PTSD	Abstinent	Diabetes	Local MH/ addiction services
Runar (m)	37	ADHD, Depression	Cannabis Heroin	Musculoskeletal disorders	Local MH/ addiction services
Philip (m)	41	Depression	Alcohol Cannabis Heroin		Local MH/ addiction services
Thomas (m)	43	Anxiety	Abstinent	COPD	Local MH/ addiction services
Jan (m)	26	ADHD	Alcohol Heroin		Local MH/ addiction services

*m* male, *f* female

### Data collection

We conducted 17 semi-structured individual interviews and one focus group interview with five participants in September and October 2020, since most participants wanted to be interviewed individually rather than in a group. The number of interviewees was based on experience from previous studies [28, 29] with the same vulnerable target group. The interviews focused on how participants experienced the first lockdown from March to May 2020. Based on our experience from previous studies [28], the first author conducted the interviews accompanied by a peer support researcher, with lived experience of SUD/MHD. Evidence suggests that peer involvement in the co-production of research ensures that the research process is more sensitive to the needs of participants [30]. A semi-structured interview guide (Table 2) consisting of open-ended questions was created in collaboration with the reference group and was used in both individual interviews and the focus group. Individual interviews lasted between 30 and 60 minutes, adjusted to the condition of the interviewees at the time, while the focus group interview lasted 90 minutes. Individual interviews took place either in the participant’s home or in rooms belonging to agencies involved in recruitment, where the focus group interview was also conducted. None of the participants took part in both the focus groups and individual interviews. All interviews were conducted in Norwegian.

**Table 2:** Semi-structured interview guide

**Initial questions**	**Experience during the lockdown**
How old are you?	How did you hear about the restrictions due to COVID-19 and how you were supposed to act?
What kind of substances do you take?	How did the pandemic and the lockdown affect your life?
Which mental health challenges did you experience?	Which measures affected you most and how?
How did it feel to get this invitation?	How could you comply with the infection control measures?
	What is your opinion about these measures?
	What are your concerns regarding the pandemic?
	What did you do during the lockdown?
**Use of health services**	**Follow-up questions**
How did you use the health/social services during the pandemic?	How did that feel?
What do you expect from health/social services during a pandemic?	What are your thoughts about that?
	Do you have any examples?
	Would you like to mention something we haven’t talked about?

### Analysis

**Fig. 1:**
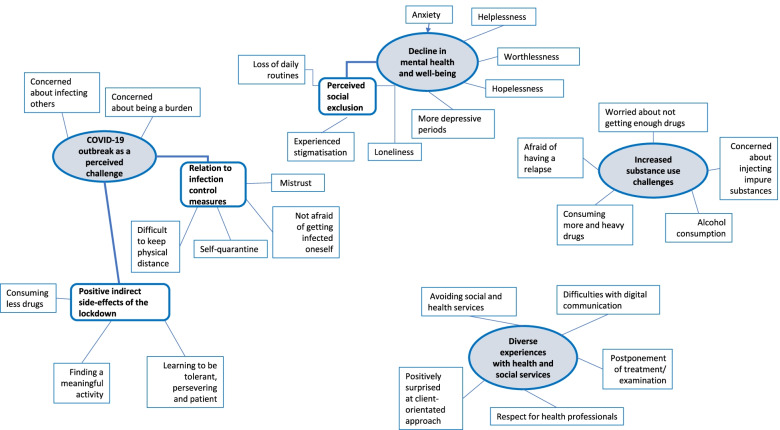
Final thematic map: generating themes and sub-themes from codes

Both individual and focus group interviews were audiotaped and transcribed verbatim. We applied Braun and Clarke’s [31] thematic analysis which comprises the following steps: (1) Familiarising oneself with the data, (2) Generating initial codes, (3) Searching for themes, (4) Reviewing themes, (5) Defining and naming themes, and (6) Producing the report. For the purpose of analysis, the first author listened to the recordings several times and read the transcripts closely for surface and underlying meaning, created codes to represent components of meaning and constructed themes by identifying patterns of meaning within and across transcripts. The first author presented the primary codes to the reference group. These codes were then reviewed and revised together with the reference group. Mind mapping was later used to group the codes into themes and to show the overall conceptualisation of the data patterns and their relationships (see Fig. 1). To reflect the nuances of the identified themes, we enriched the results section with quotes translated from Norwegian, where participants have been given pseudonyms with their real age in brackets.

### Ethical considerations

Persons living with MHD/SUD are considered a vulnerable group. Certain topics during the data sampling process may have upset some members of the group. Therefore, follow-up activities and support were provided to minimise any harm. On balance, the greater risk of not interacting with individuals and groups that have been underrepresented in clinical research needs to be recognised, as it prevents their voices from being heard and their knowledge from being incorporated into strategies for recovery. We included only persons who could read the information letter in Norwegian and who could provide informed consent prior to participation. We applied ethical procedures to incorporate autonomy, empowerment and to reduce risks at each stage of the project. Details that could identify participants were removed before the material was shown to the reference group. Participants were offered the opportunity to get in touch with the researchers and the peer support workers after the interviews. Ethical approval for the entire project was obtained from the South-Eastern Regional Committee for Medical and Health Research Ethics (reference number 158909) and from the data protection office of Innlandet Hospital Trust (reference number 135540).

## Results

Four interrelated themes were identified in the analysis, describing how the first lockdown affected the everyday life and health of persons suffering from MHD and SUD: 1) The COVID-19 outbreak as a perceived challenge, 2) A decline in mental health and well-being, 3) Increased substance use challenges, and 4) Diverse experiences with health and social services. These themes are presented with their respective sub-themes.

### Theme 1: The COVID-19 outbreak as a perceived challenge

All interviewees described the lockdown in connection with the COVID-19 outbreak as a burden and a significant challenge in their lives. The participants reported having several concerns and worries due to the pandemic. The possibly of infecting others was a great concern to everyone. They were especially afraid of infecting older people and relatives, since they felt that they were more vulnerable to COVID-19 than others and did not want to be transmitters of the disease:

“*I started coughing and then I was afraid I’d got it, locked myself in for two weeks and I was so scared I’d infected them* (the family).” *(Marius, 39)*

Some said that they paid special attention to avoid infecting their loved ones:

“*I didn’t see my children because I wanted to pay attention to them, I don’t want them to get infected because I live in a place like this* (supported housing).” *(Kari, 56)*

Another concern was being a possible burden to society if they became infected. Participants said that they did not want to bother anyone, because they felt that other people needed more help than they did themselves. One woman put it like this:

“*I could have died instead of many others who’ve died, I’m just an old drug addict.*” *(Hilde,60)*

On the other hand, almost none of the participants were afraid of becoming infected themselves, as they didn’t know anyone who had been infected with COVID-19. Another explanation they had for the low prevalence of COVID-19 among persons who consume drugs under the first lockdown was that *“… maybe the dope kills the virus.” (Jan, 26)*

Some believed that drug addicts are somehow immune to COVID-19:

*“I’m not worried about getting ill, maybe addicted people are more cautious than others.”*

“… *probably a lot of toxins in me that drive out the coronavirus.*” *(Thale, 60)*

#### Relation to infection control measures

With regard to how the informants handled the lockdown, the results are ambiguous. Some participants found strategies, such as putting oneself in quarantine, sleeping a lot or watching TV all day long, while those suffering from anxiety or severe depression, struggled to cope with even a single day. The majority reported having an understanding for the infection control measures. Most of them insisted that they followed the rules such as washing and disinfecting their hands, maintaining physical distance and having little contact with others to avoid infection:

“*Those rules are for our own good. I follow the rules, but there are so many people who don’t. Because you’ll never be careful enough, so I feel like what I’m doing is what I can do to keep COVID away from me and my friends.*” *(Kari,56)*

Most informants imposed self-quarantine on themselves and stayed indoors for the first three weeks of the lockdown. They felt it was the only thing they could do to avoid infecting others and coping with the new circumstances of the pandemic. Many experienced this self-quarantine as a state of emergency but also the only way to handle the early stage of the pandemic. Unlike the majority, one informant admitted that he tried to ignore the pandemic by living life as usual:

“*I’m in contact with all my friends, we don’t care, we hang around and use the same pipe, and none of us have been infected with COVID so far.*” *(Jan, 26)*

Another participant, an active drug user, showed mistrust:

“*I don’t trust the statistics, up and down and to and from. I think that’s not real. That helps me.*” *(Philip,41)*

Jan, who was hanging out with his friends, found that ignoring the virus was the best way of handling the lockdown: “*Nobody’s going to tell me what to do or not to do. I know myself what’s best for me.*” *(Jan, 26)*

 Participants noted that keeping physical distance to others was difficult. Social gestures such as shaking hands and hugging were mentioned by both those who were abstinent and those using substances as an important part of social routines that usually improved their well-being. Avoiding physical contact was a great challenge for both women and men:

“*Physical contact is important, it’s just as important for people in the drug scene as for everyone else. Also, the sexual bit is no longer a topic since COVID.*” *(Thomas, 43)*

Some found themselves in a dilemma, balancing the advantages and risks of hugging a person, which was stressful:

“*You have to make a choice if the hug is worth the risk, it drives me crazy.*” *(Tuva, 46)*

#### Positive indirect side-effects of the lockdown

A third of the participants, i.e. those who were either abstinent or consumed legal substances, like alcohol and tobacco, found out that a meaningful activity was helping them to cope with the lockdown. Some started reading books, going for walks, listening to music or playing the guitar. Several informants said that there was also something positive about the lockdown. A woman in her 50s explained proudly that she had learned to enjoy her own company. Another informant reported that taking up guitar playing made him take less drugs at a time when society was shut down:

“*I played the guitar a lot because I wasn’t much with other people and drank less and smoked less because I socialised less*.” *(Vegard, 38)*

Others mentioned that they took substances less during the first lockdown since there was little socialising with others due to the national restrictions, which they found positive. These people usually used substances while going out with peers.

### Theme 2: A decline in mental health and well-being

The participants who could not find any benefit from the pandemic, which was the majority, reported having more periods of depression and anxiety during the first COVID-19 lockdown. A man in his 30s using amphetamines explained that he began self-medication due to increasing depressive episodes.

“*In the beginning I was scared, thought it was the Black Death, but I’m not afraid of dying, I think it’s just really frightening. I’m living in constant fear, taking some pills to get rid of these suicidal thoughts.*” *(Espen, 31)*

Another man who lived in supported housing described how he would spend long periods just lying in his room, becoming more and more depressive. Mari, a woman on an OST programme, stated that her greatest challenges were tremors triggered by her concerns about the pandemic. Feeling hopeless in general and being powerless against the virus was reported to have a negative impact on participants’ mental health.

#### Perceived social exclusion

Participants living alone in council housing were afraid of increasing social isolation, if society was shut down for a long time. It also emerged that most interviewees felt lonelier during the lockdown, although some found little difference from pre- pandemic times.

Further, many interviewees reported experiencing stigma due to their MHD/SUD. A woman mentioned an incident in a supermarket:

“*People look at you especially if you obviously look like a drug addict. Someone asked me if I was a super spreader. I got so angry.*” *(Tuva, 46)*

Another woman found that people kept greater distance from her and referred to a conductor on the train who ignored her when she wanted to show her tickets, but checked the tickets of the other passengers. This was a humiliating experience for the informant and made her feel particularly stigmatised during COVID-19. Moreover, the loss of daily routines and structures was described as challenging for the mental health for most informants. Some were engaged in a work programme that was affected by the lockdown in March 2020. Either the programme was suspended, or the work could not be carried out as usual due to the governmental infection control regulations. A participant who normally sold a charity magazine in the pedestrian zone explained:

“*When corona came, no one would buy any more magazines from me. Now I have nothing to do. I miss my work routines. It’s so meaningless.*” *(Kevin, 47)*

For Kevin this situation not only implied a loss of daily routine and income but he also experienced discrimination. Kevin gave the impression that nobody wanted to buy magazines from a drug addict who might spread the virus easily and said that made him feel worthless. Another participant used to clean toilets regularly, but the national lockdown put the job on hold. Although the informant said that this was not a prestigious job, he felt that it was meaningful, and the loss of this activity made him feel worthless. Further, the closed soup kitchens, meeting places and facilities aimed at persons with SUD were mentioned as affecting the mental health of the participants. Although later in the lockdown there were alternatives, such as ‘walk and talk’ (one-to-one counselling while going for a walk) or the distribution of food packages, the interviewees said that it was challenging to adapt to the new measures and they still felt distressed. Harald, a man who usually went to a soup kitchen where he could also take a shower twice a week, reported that he had no other place to go when this facility was shut down. This made him feel helpless and anxious.

Losing their social networks was a concern expressed by many participants, especially those who reported being in a recovery process. Peter explained that he had spent considerable time building his self-confidence, which helped him to join a group of Narcotics Anonymous. This network helped him to become drug-free.

“*I was afraid of losing the platform I’d built up after I’ve been drug-free, it was frightening.*” *(Peter, 50)*

Little contact with other people and loss of daily routines were also grounds for concern among those who were mentally stable, as they were afraid of having a relapse of a mental health disorder.

### Theme 3: Increased substance use challenges

Being lonely without a social network meant that people who were cutting down on substances or had become substance-free were afraid of a relapse. Others said they were tempted to buy alcohol or drugs to allay their fear of being alone:

“*I was clean when COVID came, I’m afraid of being tempted to take more dope.*” *(Peter, 50)*

However, the informants who reported using illicit drugs said that they started to take drugs more frequently or in stronger doses than usual, otherwise they would not have been able to tolerate the COVID-19 situation. A woman who had stopped taking heroin became addicted again:

“*I started messing with heroin in May, needed to relax a little bit.*” *(Elisabeth, 34)*

Further, these users reported that there was a short period early in the pandemic when cannabis and heroin were not as easily available as usual. Along with this, there was a concern among the illicit drug users that the heroin might be diluted and thus contaminated, due to the shortage of supply.

Another informant mentioned that getting high was the only thing that helped him to get through the pandemic:

“*The biggest challenge was getting enough drugs. I needed something stronger than what I was used to.*” *(Bjørn, 41)*

Some men stated that they drank until they did not feel anything anymore, while two women mentioned that they took amphetamines for anxiety. Getting drunk or high was described as self-medication, a way to handle the COVID-19 situation.

### Theme 4: Diverse experiences with health and social services

Overall, most participants reported having had less contact than usual with health and social services, or they had no contact at all during the first month of the pandemic. Several informants found that scheduled appointments with a doctor were postponed or cancelled. This applied also to persons who had an additional physical disorder. Two participants had planned to go to a detoxification facility, but their admission was postponed. However, the majority expressed complete understanding for these postponements. Some were offered a digital consultation as an alternative to a physical appointment, but this did not suit everyone:

*“Because of my COPD I was going to the doctor but got no appointment. Then I was offered a video consultation but I refused it and then I never spoke to my GP again.” (Jens, 57)*

This also applied to another informant who described his experience with the local mental health centre: “*It was shut down, but then we used video calls. I didn’t like it, it didn’t feel real, but I did it anyway.*” *(Runar, 37)*

Furthermore, contact with social services such as the national public welfare agency (NAV) was perceived as challenging. It was mentioned several times that it was difficult to get in touch with these services as physical attendance was no longer possible. Some felt devalued because they felt they did not have enough digital expertise. One said:

“*NAV is absolutely hopeless, just a digital post box, nobody picks up the phone, I feel so powerless, discouraged. I depend on these disability benefits.“(Steffen, 45)*

Informants who were in an OST programme had the impression that health professionals who supplied medications were more distanced and busier at the beginning of the lockdown. They stated that the methadone was delivered on their doorstep and that nurses avoided going to clients’ homes. Participants also mentioned that the delivery times of the substitution drugs varied greatly, which was challenging for some of them.

“*I get a methadone delivery every day. But they’re not coming in. I understand that they have a lot to do, but if they don’t come on time, they mess up my day.*” *(Mari, 47)*

All informants who were in an OST appreciated being able to continue in the programme even during the pandemic.

Half of the informants who were in contact with health and social services showed an understanding for how health professionals acted during the pandemic. They explained that they gained respect for nurses and doctors who exposed themselves to a possible COVID-19 infection.

 Two participants were positively surprised to receive a call from their GP to ask how they were doing. They felt that the pandemic made health professionals more attentive:

“*My doctor just showed up at the door and asked how I was doing. We sat outside and talked; it was good to be seen as a person who needs special attention.*” *(Harald, 43)*

The council addiction services also received a positive mention when they contacted participants proactively by phone and provided walk and talk consultations.

## Discussion

The purpose of this analysis was to investigate how the first COVID-19 lockdown in Norway affected the everyday life and health of persons with MHD/SUD. Findings show that the early phase of the pandemic affected all aspects of life in this group; effects were mostly negative, but also positive in certain ways. Participants experienced increasing mental health challenges and faced further challenges related to substance use, more loneliness, greater anxiety, and feelings of stigmatisation, while some established new strategies to face the lockdown. Participants generally found that they could not access health and social services as before; although some were successful, several others faced challenges. The apprehensions of the participants often involved concern for others rather than themselves. It was common for persons with MHD and SUD to be afraid of potentially infecting others with the virus and thus contributing to more suffering. These worries about becoming a burden for society in case they infect someone is an expression of altruistic behaviour but is also linked to the fear of confirming already existing prejudice about persons with MHD and SUD, such as that their disorder is self-inflicted, and that they lack self-discipline and willpower [32–34]. Another explanation can be that persons with mental health problems tend to internalise stigmatising attitudes present in the public domain, [35]. Factors such as loneliness, the dilemma between the urge for physical contact and the recommendation to maintain physical distance, and stigma and social exclusion seemed to provoke their mental health problems and lead to longer and more intense periods of anxiety and depression. Other studies have identified a high prevalence of anxiety and depression among the general population during COVID-19 [36–40]. If the pandemic leads to poorer mental health outcomes among the general population, it might be even worse for persons with already existing MHD and SUD. The majority of our study population lived alone, either in council housing or supported accommodation. Thus, family and friends were not present and due to government restrictions, reaching out to peers was difficult during the first lockdown. Social distancing and the loss of daily routines may help to explain the decline in mental health among our target group. Moreover, some informants reported having difficulty complying with the requirements to self-isolate or maintain physical distance from others. This may also have served as a type of overdose prevention strategy for those who were consuming opioids, avoiding using substances alone. Others, studying the situation during and after COVID-19 lockdown found an increasing prevalence of SUD in the general population and a higher risk of multi-morbidity and mortality among persons with opioid use disorders [41, 42].

Our study confirms the experience of stigmatisation during the pandemic. This not only applied to persons with MHD/SUD but also to other groups such as immigrants, persons who recovered from COVID-19 or those with a low socioeconomic status [43]. Stigma during COVID-19 may be comparable to stigma and discrimination due to other diseases such as HIV and tuberculosis [44]. The results suggest that persons with MHD and SUD are exposed to double stigma: struggling with substance abuse and mental health is often the starting point for stigma. However, they are also often portrayed in the media as being tired, filthy, and with a blurry look in search of the next shot [45]. This may lead to thinking that in a pandemic, when hygiene is especially important, persons with MHD/SUD are predestined to spread the virus. Therefore, it is important to educate society and health and social care staff to establish anti-stigma measures, especially during a pandemic such as COVID-19.

Our informants developed coping strategies, in both negative and positive directions. More consumption of alcohol or illicit drugs was among the most frequently mentioned strategies to get through the first lockdown. This is in line with the report of the European Monitoring Centre for Drugs and Drug Addiction from June 2020, based on an online survey of drug use in 21 European countries [42]. To get drunk or high and try to continue living life as usual can be one way, and ignoring the pandemic another strategy, to cope with a state of emergency like COVID-19. However, drinking more during the pandemic did not apply to all participants; a few reported drinking less, due to fewer social occasions during the lockdown. These findings are confirmed by Bramness et al., who found that almost a third of their study population, which comprised Norwegians above 18 years form the general population, drank less during the pandemic [46]. Been et al., who analysed illicit drugs in wastewater in seven European cities, presents a heterogeneous picture of illicit drug use during the first lockdown. While in some cities the consumption of stimulants decreased, greater use of drugs was seen in other cities in 2020 than in previous years [47].

A recently published literature review shows that people with substance abuse disorder are at greater risk of poorer COVID-19 outcomes [48]. The authors point out that persons who suffer from addiction have difficulty accessing health services, which makes them vulnerable and tempted to acquire drugs illegally as a form of self-medication. That is partly consistent with our results, as informants reported consuming more illicit drugs or treating mental disorders themselves by taking drugs to cope with their challenges during the pandemic. Some of the informants used the lockdown for beneficial activities, such as reading books, playing the guitar, or going for walks. These may also be types of coping strategies, as pleasant and enjoyable activities provide meaning and help to structure the day [49]. However, the majority of our study population were unable to benefit from the pandemic.

In the early phase of COVID-19, most societies were afraid that health services would collapse due to the high number of infected persons who needed care. Our findings show that persons with a MHD/SUD used social and health services less often than usual. They believed that others needed the help of social and health care providers more than they did themselves and wanted to avoid being a burden to society. This feeling of guilt and worthlessness which is common among persons with MHD and SUD [50] may make them avoid seeking out health and social services. Further, these feelings and behaviour may also have been exacerbated by the manner in which the infection control measures have been communicated by the media and the government. Since the beginning of the COVID-19 pandemic there has been an overwhelming communication of rules, regulations and recommendations and many individuals do not know how to assess critically the information they receive and implement this into concrete actions [51]. Furthermore, our study population faced barriers in contacting or having a consultation with social and health services due to their low level of digital skills. The COVID-19 pandemic has driven a growth in telemedicine, including consultations via video or chats [52]. However, this does not suit every patient group. Online consultations postulate a certain level of health literacy, digital competence and equipment such as a smartphone or computer, which cannot be taken for granted among persons with MHD and SUD, as some of our informants reported. Low to moderate health literacy levels among persons with MHD or SUD are quite common, according to Degan et al. [18].

Postponement of medical treatment, especially places in detoxification facilities, has left persons with MHD and SUD vulnerable to unplanned negative effects such as deviations in substance use behaviour, deteriorated mental health outcomes, and greater risk for virus exposure. Further, low-threshold measures such as leisure activities, work programmes and food banks especially aimed at this target group were limited and physical meetings with social and health professionals were reduced, posing major challenges for persons with MHD/SUD. These factors underline the need for continuous access to treatment, care and support services and show that cutbacks in the capacity of these services is not suitable to address the needs of persons with MHD and SUD during a pandemic. These persons depend on continuous access to health and social services. Persons with a concurrent mental health and substance use disorder face great challenges due to several factors that place them at risk of coronary artery infection and complications [26], as well as being particularly affected by the restrictions and changes in the provision of health and social services, as our study indicates. These factors probably have an additive effect and may lead to further marginalisation. There is no doubt that this pandemic is creating challenges all over the world, but also no doubt that it exacerbates that already exist. To provide appropriate services, care and treatment to this group, it is important that service providers collaborate to prevent barriers to accessing social and health services from increasing or even arising. It is also vital to maintain the structures and routines that service users are accustomed to. Ideally, these services should remain open, increase their resources and ensure access through innovative methods such as support from peer support workers and mobile outreach access [53]. These approaches might alleviate both the exposure to COVID-19 and the negative effects of lacking support.

Finally, the results of this qualitative study form a basis for the quantitative part of the project “The impact of the COVID-19 outbreak on persons with MHD and SUD” in which we will analyse register data, studying risk factors for COVID-19, the prescription of psychotropic drugs and health care utilisation by this target group.

### Strengths and limitations

This study includes data from 17 in-depth interviews and one focus group (*n* = 22 participants), which clearly contributes to the literature on the impact of COVID-19 on a vulnerable group such as persons with MHD and SUD. The interviews were conducted face-to-face, even during the restrictions due to the pandemic, which is a strength of this study. Persons with MHD/SUD belong to a vulnerable group whose mood and conditions changes from day to day and is often not predictable. A face-to-face interview was in this case a benefit, as the researcher could adapt the interview situation to the individual needs. This would not have been possible within a telephone interview [54]. A further strength is that we conducted the interviews promptly after the end of the first lockdown, which gave us fresh insight into the everyday life of the target group and reduced recall bias among our informants. Involving persons with lived experience with MHD and SUD (peer support workers) in the study design and analysis enriched the research process and minimised potential interpretation bias. The peer support workers contributed with unconventional ways of raising questions and interpreting findings and participated in an interactive arena for mutual support. However, some limitations have to be mentioned. We interviewed 22 people with self-reported MHD and SUD in one local authority in Eastern Norway. Services for the target group will vary between local authorities; elsewhere, the situation may be different. Consequently, the findings cannot be generalised to all persons with MHD and SUD, which is related to the nature of qualitative research [55]. This study focussed on the impact of the first lockdown on service users. However, it is also important to study the perspectives of persons providing mental health and addiction services, to gain an understanding of how they experienced the impact of the early stage of the pandemic on persons with MHD and SUD.

### Practical Implications

Based on the descriptions of the informants’ challenges but also positive aspects of their everyday lives during COVID-19, this study demonstrates a need to maintain low-threshold services such as meeting places, walk and talk consultations, leisure activities and work assistance continuously during the pandemic. Furthermore, it is important to enhance the digital skills of every service user. Some people may have such skills and are comfortable with a digital consultation, but the study showed that most people with MHD and SUD have challenges with digital tools and/or do not have the equipment to participate in a video consultation. Therefore, it is recommended to offer alternatives to digital consultations and meetings.

## Conclusions

The findings provide a meaningful contribution to the limited research on persons with concurrent mental health and substance use disorders in relation to the COVID-19 pandemic. There is reason to believe that new pandemics or other situations that threaten the population will emerge in the future. In this context, it is essential to gain knowledge of how to care for vulnerable groups in society and how to reach them in emergencies. This knowledge will ensure that persons with MHD/SUD are not discriminated against if a rise in cases of COVID-19 places an additional burden on the health care system.

## Data Availability

The anonymised transcripts used during the current study are available from the corresponding author on request.
